# Kapur’s Entropy for Color Image Segmentation Based on a Hybrid Whale Optimization Algorithm

**DOI:** 10.3390/e21030318

**Published:** 2019-03-23

**Authors:** Chunbo Lang, Heming Jia

**Affiliations:** College of Mechanical and Electrical Engineering, Northeast Forestry University, Harbin 150040, China

**Keywords:** Kapur’s entropy, color image segmentation, whale optimization algorithm, differential evolution, hybrid algorithm, Otsu method

## Abstract

In this paper, a new hybrid whale optimization algorithm (WOA) called WOA-DE is proposed to better balance the exploitation and exploration phases of optimization. Differential evolution (DE) is adopted as a local search strategy with the purpose of enhancing exploitation capability. The WOA-DE algorithm is then utilized to solve the problem of multilevel color image segmentation that can be considered as a challenging optimization task. Kapur’s entropy is used to obtain an efficient image segmentation method. In order to evaluate the performance of proposed algorithm, different images are selected for experiments, including natural images, satellite images and magnetic resonance (MR) images. The experimental results are compared with state-of-the-art meta-heuristic algorithms as well as conventional approaches. Several performance measures have been used such as average fitness values, standard deviation (STD), peak signal to noise ratio (PSNR), structural similarity index (SSIM), feature similarity index (FSIM), Wilcoxon’s rank sum test, and Friedman test. The experimental results indicate that the WOA-DE algorithm is superior to the other meta-heuristic algorithms. In addition, to show the effectiveness of the proposed technique, the Otsu method is used for comparison.

## 1. Introduction

Image segmentation is a fundamental and key technique in image processing, computer vision, and pattern recognition, the purpose of which is to partition a given image into specific regions with unique characteristics and then extract the objects of interest [1,2,3,4]. Hence, the segmentation technique to be adopted determines the performance of higher level systems that introduced above [5]. At present, the main techniques of image segmentation include edge-based technique, region-based technique, neural network-based technique, wavelet transform-based technique, and threshold-based technique [6,7,8,9,10]. Among the available techniques, threshold-based technique (thresholding) is the most popular one that many scholars have done much work in this domain.

More specifically, the thresholding technique determines the segmentation thresholds by optimizing some criteria, such as maximum between-class variance and various entropy criteria [11]. In 1985, Kapur et al. maximized the histogram entropy of segmented classes to obtain the optimal threshold values, which is known as Kapur’s entropy technique [12]. This thresholding technique is adopted extensively and show remarkable performance in many image segmentation problems. However, when dealing with complex image segmentation problem, the high threshold operation will increase the computational complexity of the algorithm significantly. Thus, scholars introduce various meta-heuristic algorithms into this domain with the view of reducing computational complexity and improving segmentation accuracy. Shen et al. [13] proposed a modified flower pollination algorithm (MFPA)-based technique for segmenting both real-life images and remote sensing images. The experimental results show that the MFPA algorithm gives higher values in terms of PSNR and SSIM, which is suitable for high dimensional complex image segmentation. In 2016, Kapur’s entropy thresholding technique was adopted by Sambandam and Jayaraman [14] for multilevel medical image thresholding. The proposed technique was then optimized by dragonfly optimization (DFO) with the purpose of reducing computational complexity. It can be seen from the results that the proposed algorithm can efficiently explore the search space and obtain the optimal thresholds. In 2017, Khairuzzaman and Chaudhury [5] proposed a grey wolf optimizer (GWO)-based technique for multilevel image thresholding. Kapur’s entropy and Otsu methods are used to determine the segmentation thresholds. Experimental results show that the GWO-based technique using both Kapur’s entropy and Otsu thresholding techniques performs better than particle swarm optimization (PSO) and bacterial foraging optimization (BFO)-based methods. Besides, there are still many other meta-heuristic algorithms have been successfully applied to multilevel image thresholding, such as artificial bee colony (ABC) [15], firefly algorithm (FA) [16], cuckoo search (CS) [17], wind driven optimization (WDO) [18], krill herd optimization (KHO) [19], moth-flame optimization (MFO) [20], etc. It is well known that the overwhelming majority of images in practical engineering problems are color images, which are often complex and contain a lot of information, whereas, most of the techniques above are used to segment the grayscale images rather than color images. This phenomenon motivated us to introduce an efficient technique to satisfy the practical requirements.

The whale optimization algorithm (WOA) is a novel meta-heuristic algorithm that simulates the behavior of humpback whales in nature [21]. There are mainly three foraging behaviors, namely encircling prey, bubble-net attacking, and search for prey. WOA is a simple and powerful algorithm that has attracted wide attention from scholars recently [22]. In 2018, Xiong et al. [23] used a WOA algorithm to extract the parameters of solar photovoltaic (PV) models. Compared to the conventional as well as recently-developed methods, the proposed algorithm can determine the parameters more accurately. Sun et al. [24] proposed a modified whale optimization algorithm (MWOA) for solving large-scale global optimization (LSGO) problems. Twenty-five benchmark test functions with various dimensions were utilized to verify the performance. The experimental results indicated that the proposed algorithm is superior to other state-of-the-art optimization algorithms in terms of accuracy and stability. In 2017, Mafarja and Mirjalili [25] introduced two hybridization models of WOA and simulated annealing (SA) and then applied the proposed methods to feature selection domain. The SA was adopted to enhance the exploitation capability. It can be observed that the proposed hybrid algorithm outperformed other wrapper-based algorithms in classification accuracy, which is suitable for the current optimization task [25]. To sum up, these promising results motivate us to introduce the WOA algorithm into color image segmentation domain.

It is worth mentioning that color image multilevel thresholding operations need to determine the thresholds of every color component (red, green, and blue), while a meta-heuristic algorithm with strong optimizing capacity can improve the accuracy of image segmentation, as it can obtain appropriate thresholds [26]. Therefore, an improved whale optimization algorithm is proposed which is known as WOA-DE. In the proposed algorithm, differential evolution (DE) is served as local search technique to enhance the exploitation ability. What’s more, introducing DE operator improves the situation that the traditional WOA is easy to fall into local optimum in the later iteration. In order to obtain an efficient and universal segmentation method, the performance of WOA-DE using Kapur’s entropy is investigated. A series of experiments are conducted on both natural images and satellite images. All experimental results are compared with state-of-the-art algorithms as well as conventional methods. It can be observed from the results that the WOA-DE based methods outperform other meta-heuristic based methods in terms of average fitness values, standard deviation (STD), peak signal to noise ratio (PSNR), structural similarity index (SSIM), feature similarity index (FSIM), and the Wilcoxon’s rank sum test as well as the Friedman test. The goal of this paper is as follows:

1. Obtain an efficient segmentation technique for multilevel color image thresholding task.
2. Improve the optimizing capability of WOA to determine the optimal thresholds.
3. Investigate the adaptability of WOA-DE based techniques in the field of natural, satellite, and MR image segmentation.
4. Evaluate the performance of proposed technique from various aspects.

The structure of this paper is presented as follows: Section 2 gives the definition of Kapur’s entropy thresholding technique. Section 3 introduces a brief review of the WOA algorithm. The description of the DE algorithm is presented in Section 4. In Section 5, the proposed WOA-DE-based multilevel color image thresholding technique is described in details. Experiments and discussion can be found in Section 6. Finally, Section 7 presents the conclusions and future work directions.

## 2. Multilevel Thresholding

The image threshold methods can be summarized into two categories: bi-level thresholding methods and multilevel thresholding methods. Bi-level thresholding methods involve one threshold value which partitions the image into two classes: foreground and background, however if the image is quite complex and contains various objects, the bi-level thresholding method is not very effective [27,28,29,30]. Therefore, multilevel thresholding methods are used extensively for image segmentation [31,32,33]. In this paper, a famous multilevel thresholding technique is used to determine the threshold values, namely, Kapur’s entropy. A brief formulation of this technique is given in the following subsections. In addition, the RGB image has three basic color components of red, green, and blue, so these thresholding techniques are executed three times to determine the optimal threshold values of each color component [16].

### Kapur’s Entropy

Kapur’s method is also an unsupervised automatic thresholding technique, which selects the optimum thresholds based on the entropy of segmented classes [12]. Assuming that $\left[th_{1},th_{2},\ldots ,th_{n}\right]$ represents the thresholds combination which divided the image into various classes. Then the object function of Kapur’s method can be defined as:

(1) $$H\left(th_{1},th_{2},\ldots ,th_{n}\right)=H_{0}+H_{1}+\ldots +H_{n}$$

where:

(2) $$H_{0}=-\sum_{j=0}^{{th}_{1}-1}\frac{p_{j}}{\omega_{0}}\ln\frac{p_{j}}{\omega_{0}},\;\omega_{0}=\sum_{j=0}^{{th}_{1}-1}p_{j}$$

(3) $$H_{1}=-\sum_{j=th_{1}}^{{th}_{2}-1}\frac{p_{j}}{\omega_{1}}\ln\frac{p_{j}}{\omega_{1}},\;\omega_{1}=\sum_{j=th_{1}}^{th_{2}-1}p_{j}$$

(4) $$H_{n}=-\sum_{j=th_{n}}^{L-1}\frac{p_{j}}{\omega_{n}}\ln\frac{p_{j}}{\omega_{n}},\;\omega_{n}=\sum_{j=th_{n}}^{L-1}p_{j}$$

$H_{0}$, $H_{1}$,…, $H_{n}$ denote the entropies of distinct classes, $\omega_{0}$, $\omega_{1}$,…, $\omega_{n}$ are the probability of each class.

In order to obtain the optimal threshold values, the fitness function in Equation (5) is maximized:

(5) $$f_{Kapur}\left(th_{1},th_{2},\ldots ,th_{n}\right)=\arg\max\left\{H\left(th_{1},th_{2},\ldots ,th_{n}\right)\right\}$$

It is worth noting that the computational complexity of the thresholding technique above will result in exponential growth as the number of thresholds increase. Under such circumstances, Kapur’s entropy method is not very effective for multilevel thresholding. Therefore, the WOA-DE-based method using Kapur’s entropy is proposed to improve the accuracy and computation speed of thresholding techniques. The ultimate goal of proposed method is to determine the optimal threshold values by maximizing the objective function given in Equation (1).

## 3. Whale Optimization Algorithm

**Figure 1:**
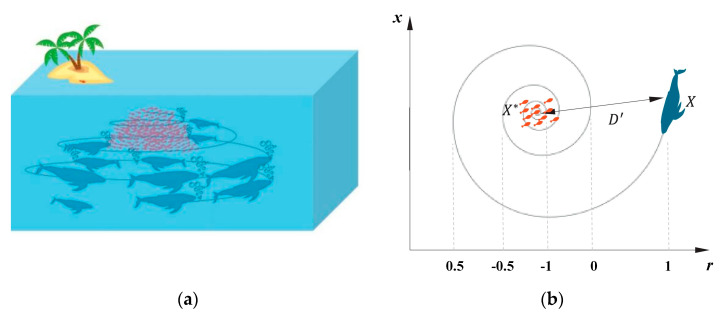
Bubble-net feeding behavior of humpback whale (**a**) and the position update model (**b**).

The whale optimization algorithm, which was proposed by Mirjalili and Lewis in 2016, is inspired by the foraging behavior of humpback whales in nature [21]. Humpback whales tend to create spiral bubbles, and then swim to the prey along the trajectory of bubbles (see Figure 1) [25]. The encircling prey and bubble-net attacking behaviors represent the exploitation phase of optimization. The other phase of optimization namely exploration is represented by the search for prey behavior. It is worth noting that the position vector of search agent is defined in a d-dimensional space, where d denotes the number of decision variables of an optimization problem. Thus, the population *X* of *n* search agents can be represented by a (*n* *× d*)-dimensional matrix, which is shown in Equation (6):

(6) $$X=\left[\begin{array}{llll} x_{1,1} & x_{1,2} & \ldots & x_{1,d}\\ x_{2,1} & x_{2,2} & \ldots & x_{2,d}\\ \vdots & \vdots & \ldots & \vdots \\ x_{n,1} & x_{n,2} & \ldots & x_{n,d} \end{array}\right]$$

### 3.1. Exploitation Phase (Encircling Prey and Bubble-Net Attacking Method)

In the process of hunting, the humpback whales first encircle the prey, which can be represented as follows:

(7) $$D=\left|C\cdot X^{*}\left(t\right)-X\left(t\right)\right|$$

(8) $$X\left(t+1\right)=X^{*}\left(t\right)-A\cdot D$$

where $X^{*}$ represents the best solution obtained so far, *X* denotes the position vector, *t* is the current iteration, || is the absolute value, · is an element-by-element multiplication, *A* and *C* are two essential parameters that can be evaluated by:

(9) A=2a⋅r−a

(10) C=2⋅r

where *r* is a random number in the range of [0,1] and *a* is a constant that will decrease linearly from 2 to 0 within the whole iterative process (both exploration and exploitation). It can be observed from Equation (8) that search agents can update their position *X*(*t*) according the best solution $X^{*}$. The parameters *A* and *C* determine the distance between the updated position X(t+1) and the optimal position $X^{*}$.

The bubble-net attacking behavior can be mathematically represented by the following equation:

(11) $$D^{\prime }=\left|X^{*}\left(t\right)-X\left(t\right)\right|$$

(12) $$X\left(t+1\right)=D^{\prime }\cdot e^{br}\cdot \cos\left(2\pi r\right)+X^{*}\left(t\right)$$

where D′ shows the distance between the current search agent position and the optimal position, *b* is a constant that determine the shape of a logarithmic spiral, and *r* is a random number in the range of [−1,1]. In order to transform these two mechanisms (encircling prey and bubble-net attacking method) of exploitation phase, assume that each mechanism will be executed with 50% probability. Thus, the mathematical model of the entire exploitation phase can be expressed as:

(13) $$X\left(t+1\right)=\left\{ \begin{array}{ll} X^{*}\left(t\right)-A\cdot D & if\;p<0.5\\ D^{\prime }\cdot e^{br}\cdot \cos\left(2\pi r\right)+X^{*}\left(t\right) & if\;p\geq 0.5 \end{array}\right.$$

where *p* is a random number in the range of [0,1].

### 3.2. Exploration Phase (Search for Prey)

In order to enhance the exploration capability of algorithm, a global search strategy is utilized. The search agents update their position according to a random agent in the population rather than the best solution obtained so far. It is worth mentioning that the absolute value of *A* determines the phase of optimization to be selected, namely the exploration and exploitation phases. Thus, the search for prey behavior can be mathematically represented as follows:

(14) $$D=\left|C\cdot X_{rand}\left(t\right)-X\left(t\right)\right|$$

(15) $$X\left(t+1\right)=X_{rand}\left(t\right)-A\cdot D$$

where *X_rand_* denotes a random individual in the current population.

Pseudo code of traditional whale optimization algorithm based multilevel thresholding has been given in Algorithm 1.

**Algorithm 1** Pseudo code of whale optimization algorithm based multilevel thresholdingInitialize the position of whales *X_i_*. Initialize the best search agent $X^{*}$. WHILE *t* < Maximum number of iterations  FOR *i* = 1:*n*   Calculate the objective value of each search agent by using the Equation (1) for Kapur’s entropy.   Update the best search agent $X^{*}$.   Update *a*, *A*, *C*, *r*, and *p*   IF1 *p* < 0.5    IF2 |*A*| < 1     Update the position of search agent using Equations (7) and (8).    ELSE     Update the position of search agent using Equations (14) and (15).    END IF2   ELSE    Update the position of search agent using Equations (11) and (12).   END IF1   Correct the position of the current search agent if it is beyond the border.  END FOR END WHILE Return $X^{*}$, which represents the optimal threshold values of segmentation.

## 4. Differential Evolution

Differential evolution (DE) algorithm is a simple and powerful algorithm for solving optimization problems [34,35,36]. Basically, the DE algorithm contains two significant parameters, namely mutation scaling factor denoted by *SF* and crossover probability denoted by *CR* [37]. For the standard DE algorithm, the mutation, crossover, and selection operators can be summarized as follows [38]:

### 4.1. Mutation Operation

The mutation operation of DE algorithm is defined as follows:

(16) $$m_{i}^{g+1}=x_{r1}^{g}+SF\times \left(x_{r2}^{g}-x_{r3}^{g}\right)$$

where $m_{i}^{g+1}$ represents the mutant individual in the (*g* + 1)-th generation. $x_{r1}^{g}$, $x_{r2}^{g}$, and $x_{r3}^{g}$ are different individuals from the population. In other words, $r_{1}$, $r_{2}$, and $r_{3}$ cannot be equal. *SF* is a constant that indicates the mutation scaling factor.

### 4.2. Crossover Operation

In the process of crossover, the trial individual $c_{i}^{g+1}$ is selected from the current individual $x_{i}^{g}$ or the mutant individual $m_{i}^{g+1}$ on account of enhancing the diversity of population. The crossover operation of DE algorithm is described as:

(17) $$c_{i}^{g+1}=\left\{ \begin{array}{ll} m_{i}^{g+1} & if\;rand\leq CR\\ x_{i}^{g} & if\;rand>CR \end{array}\right.$$

where *rand* represents a random value which is in the range [0,1]. *CR* is a constant that shows the crossover probability.

### 4.3. Selection Operation

After the process of selection, the individual of next generation $x_{i}^{g+1}$ is selected according to the comparison of fitness value between the trail individual $c_{i}^{g+1}$ and the target individual $x_{i}^{g}$. For a problem to be minimized, the selection operation of DE algorithm can be summarized as follows:

(18) $$x_{i}^{g+1}=\left\{ \begin{array}{ll} c_{i}^{g+1} & if\;f\left(c_{i}^{g+1}\right)<f\left(x_{i}^{g}\right)\\ x_{i}^{g} & otherwise \end{array}\right.$$

where *f* denotes the fitness function value of a given problem.

## 5. The Proposed Method

**Figure 2:**
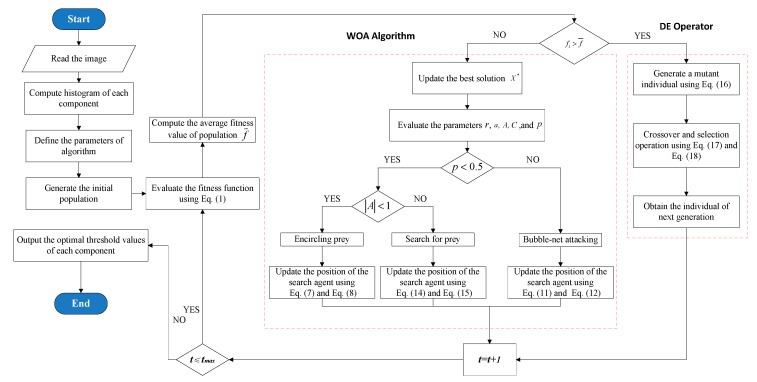
Framework of the WOA-DE based method.

In this section, a detailed introduction of the WOA-DE-based method is given, and the algorithm will be used to obtain the optimal threshold values for image segmentation. A hybrid of the WOA and DE algorithms is introduced to balance the two essential phases of optimization, namely exploration and exploitation. The flowchart of WOA-DE for finding the optimal threshold values is shown in Figure 2.

It is worth mentioning that a better balance between exploration and exploitation plays an important role in improving the optimization ability of algorithm. Therefore, an efficient hybrid strategy is introduced to balance and improve these two phases. On the one hand, the WOA algorithm has strong ability to explore the solution space and is used as global search technique. On the other hand, the DE algorithm is adopted as local search technique, which can increase the precision of solutions.

**Figure 3:**
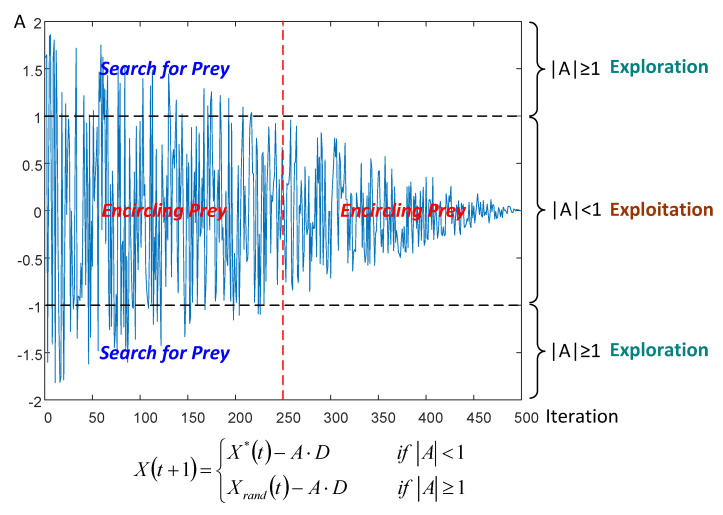
Schematic diagram of the change in random variable *A*.

In addition, the purpose of introducing DE operator is not only to enhance the local search ability of the algorithm, but also to overcome the drawback that WOA algorithms easily fall into local optima in the late iterations. As described above, the random variable *A* will change in the range [−2,2] as *a* decreases progressively. If the value larger than 1 or less than −1, Equation (15) will be adopted to enhance the exploration capability of the algorithm. On the contrary, Equation (8) will be adopted as local search strategy when the value in the range [−1,1]. In order to more intuitively reflect the change of random variable *A* during the whole iterative process, a relevant schematic diagram is presented in Figure 3. It can be observed from the figure that the value of random variable *A* is fixed in the interval of [−1,1] after 250 iterations. This means that the global search strategy has no chance to be adopted after half of the iterative process, even if the current best solution may not the global optimum. Therefore, the traditional WOA algorithm will fall into the local optimum, resulting in an unsatisfactory solution accuracy. Especially for complex multi-dimensional optimization problems, such as multilevel color image segmentation, traditional WOA algorithms cannot handle them. On the contrary, DE operators can scale the difference between any two search agents in the population, which makes the particles jump out of the current search area. In Equation (16), $\left(x_{r2}^{g}-x_{r3}^{g}\right)$ can be considered as the difference between two individuals, and SF is the scaling factor. The latter term in Equation (16) “$SF\times \left(x_{r2}^{g}-x_{r3}^{g}\right)$” is crucial to the mutation operator. For the exploration stage, particles tend to be very far apart, and there is a big difference between the individuals. Scaling this big difference can enhance the diversity of population. For the exploitation stage, particles tend to be close together, scaling a small difference makes the algorithm effectively optimize in a small range, improving the accuracy of the solution and avoiding local optimum.

In this paper, the average fitness value of the population is computed in the iterative process to evaluate the quality of each particle. The proposed hybrid model enables particles with better quality to exploit the current promising area to ensure the convergence speed, while the particles with poor quality can explore the unknown area to prevent local optimization. Although the global search strategy of traditional WOA algorithm will not be adopted in the later iteration, the introduced DE operator can effectively overcome this shortcoming, as discussed above. Exactly speaking, if $f_{i}>\bar{f}$, the DE algorithm will be used to update the solution $x_{i}^{g}$ using Equations (16)–(18). However, if $f_{i}\leq \bar{f}$, then the current solution will be updated using Equations (8), (12), or (15). In addition, a series of experiments are conducted in the following section to verify the advantages of WOA-DE algorithm from various aspects.

## 6. Experiments and Results

### 6.1. Experimental Setup

**Figure 4:**
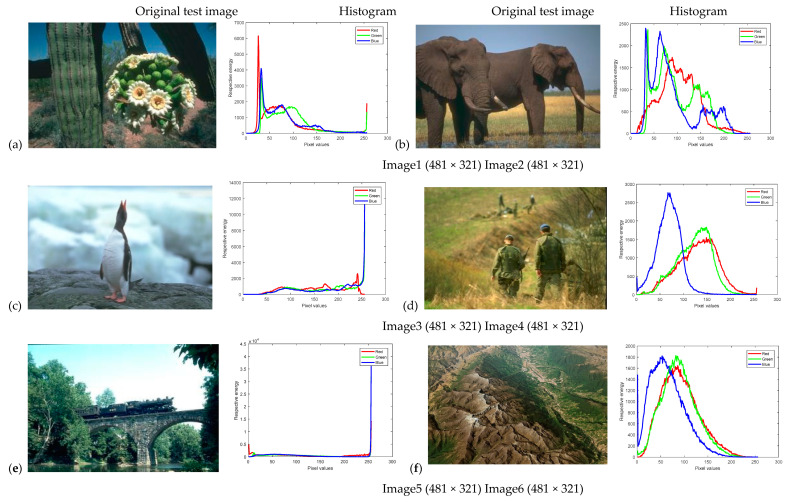

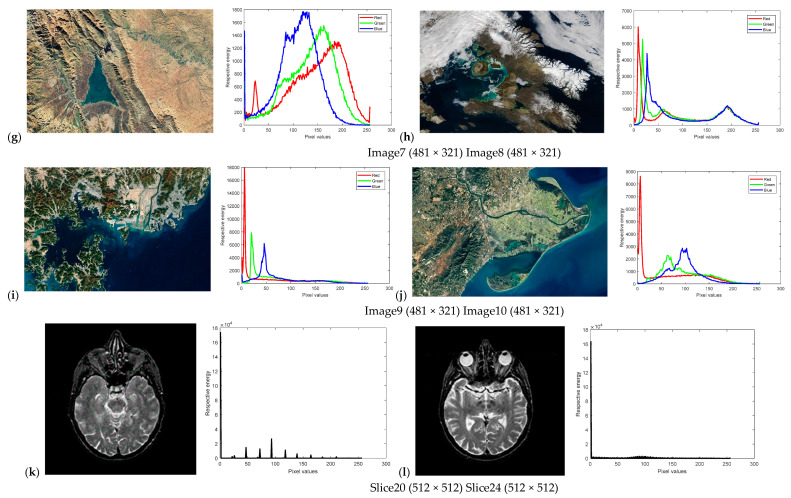

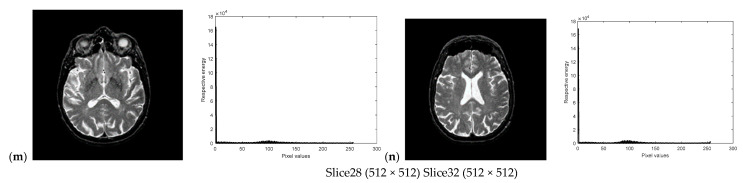
Original test images and the corresponding histograms.

In this paper, Kapur’s entropy thresholding technique is utilized to determine the optimal threshold values for image segmentation. The performance of our WOA-DE-based method is evaluated on fourteen images. Among them, five images are natural images from the Berkeley segmentation database [39], five images are satellite images from [40], and four images are brain magnetic resonance images (MRI) from [41]. Besides, all the images and their corresponding histogram images are shown in Figure 4. Both state-of-the-art and conventional methods, such as the traditional WOA [21], salp swarm algorithm (SSA) [42], sine cosine algorithm (SCA) [43], ant lion optimizer (ALO) [44], harmony search optimization (HSO) [45], bat algorithm (BA) [46], particle swarm optimization (PSO) [47,48], betaDE (BDE) [49], and improved differential search algorithm (IDSA) [50] are used to validate the superiority of proposed algorithm, whose parametric settings are presented in Table 1, except for the population size N set to 30 and the number of iterations $t_{\max}$ set to 500 for fair comparison. The experiments are carried out through the simulation in “Matlab2017” (The MathWorks Inc., Natick, MA, USA) and implemented on a computer equipped with the Microsoft Windows 10 operating system and 8 GB memory space.

### 6.2. Objective Function Measure

**Figure 5:**
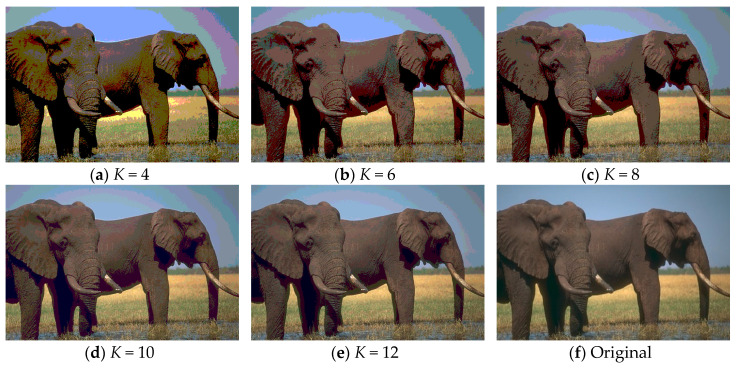
The segmented results of “Image2” at different threshold levels obtained by WOA-DE-Kapur.

**Figure 6:**
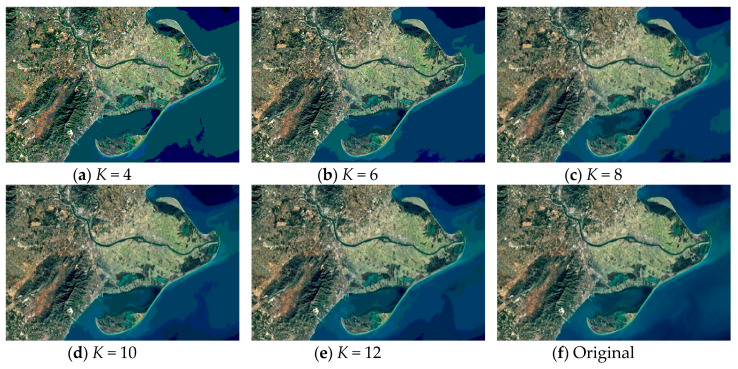
The segmented results of “Image10” at different threshold levels obtained by WOA-DE-Kapur.

As discussed above, Kapur’s entropy is used to determine the segmentation thresholds. The segmented images of “Image2” and “Image10” obtained by WOA-DE using Kapur’s entropy method with different threshold levels are given in Figure 5 and Figure 6, respectively. Due to the stochastic nature of meta-heuristic algorithms, the experiments are conducted over 30 runs. Then the average objective values of “Image1” and “Image6” are presented in Table 2. It can be seen from the table that the WOA-DE based method gives the best values in general.

The entropy of an image reflects its average information content [51]. Therefore, higher value of Kapur’s entropy indicates more information in the image. It can be observed from Table 2 that the objective function value of each algorithm increases with the number of threshold values. This promising result shows that high-quality image with more information is obtained when the threshold level is high (such as *K* = 10 and 12).

### 6.3. Stability Analysis

Standard deviation (STD): a value indicates the dispersion of sample data and it is mathematically represented as:

(19) $$STD=\sqrt{\frac{1}{n-1}\sum_{i=1}^{n}{\left(f_{i}-\bar{f}\right)}^{2}}$$

where *n* is the sample size, *f_i_* is the fitness value of the *i*-th individual, and $\bar{f}$ indicates the average value of the sample.

In order to verify the stability of proposed algorithm, the STD indicator is also used. A lower value of STD indicates better stability. The STD values of “Image1” and “Image6” obtained by all algorithms are presented in Table 2. From the table it is found that WOA-DE based method gives lower values as compared to other algorithms, which shows the better consistency and stability of proposed algorithm.

### 6.4. Peak Signal to Noise Ratio (PSNR)

Peak signal to noise ratio (PSNR): an index which is used to evaluate the similarity of the processed image against the original image [13]:

(20) $$PSNR=10\log_{10}\left(\frac{255^{2}}{MSE}\right)$$

MSE represents the mean squared error and is calculated as:

(21) $$MSE=\frac{1}{MN}{\sum_{i=1}^{M}\sum_{j=1}^{N}\left[I\left(i,j\right)-K\left(i,j\right)\right]}^{2}$$

where *I*(*i*, *j*) and *K*(*i*, *j*) denote the gray level of the original image and the segmented image in the *i*-th row and *j*-th column, respectively. *M* and *N* denote the number of rows and columns in the image matrix, respectively. A higher value of PSNR indicates a better quality segmented image.

**Figure 7:**
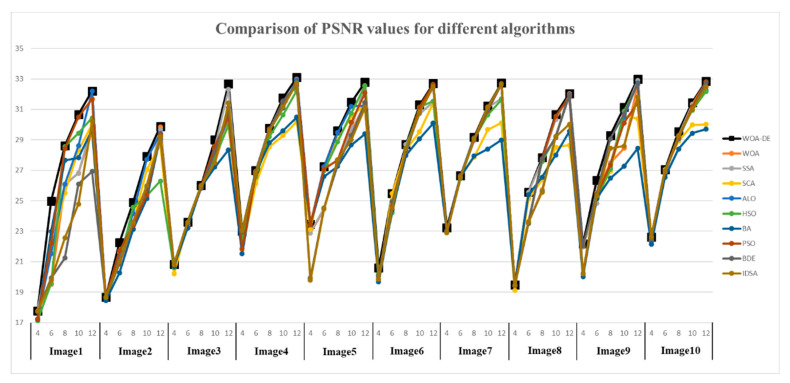
Comparison of PSNR values for different algorithms using Kapur’s entropy at 4, 6, 8, 10, and 12 levels.

Table 3 shows the PSNR values of “Image2” and “Image7” obtained by all algorithms and Kapur’s entropy method. According to the table, the WOA-DE-based method gives the highest values in 9 out of 10 cases using Kapur’s entropy. When the threshold level is small, all algorithms give similar result, while the obtained values become different as the number of thresholds increases, and the proposed method can present the best result in most cases. This phenomenon indicates that WOA-DE-based method can determine the appropriate thresholds and then present high-quality segmented image that are more similar to the original image. Figure 7 shows the visual comparison of all available methods at different threshold levels. The results of proposed method are represented as “black” lines and “square” data points.

### 6.5. Structural Similarity Index (SSIM)

Structural similarity index (SSIM) [52,53]: a measure of the similarity between the original image and the segmented image, which takes various factors such as brightness, contrast, and structural similarity into account:

(22) $$SSIM\left(x,y\right)=\frac{\left(2\mu_{x}\mu_{y}+c_{1}\right)\left(2\sigma_{xy}+c_{2}\right)}{\left(\mu_{x}^{2}+\mu_{y}^{2}+c_{1}\right)\left(\sigma_{x}^{2}+\sigma_{y}^{2}+c_{2}\right)}$$

where $\mu_{x}$ and $\mu_{y}$ denote the mean intensities of the original image and the segmented image respectively. $\sigma_{x}^{2}$ and $\sigma_{y}^{2}$ are the standard deviation of the original image and the segmented image respectively. $\sigma_{xy}$ denotes the covariance between the original image and the segmented image. $c_{1}$ and $c_{2}$ are constants. The value of SSIM is in the range [0,1], and a higher value shows better performance.

**Figure 8:**
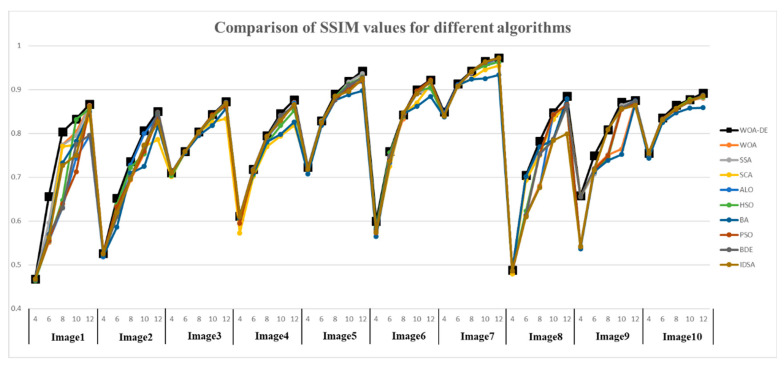
Comparison of SSIM values for different algorithms using Kapur’s entropy at 4, 6, 8, 10, and 12 levels.

The SSIM values obtained by all algorithms are given in Table 3 and Figure 8, respectively. It can be seen from the table that the WOA-DE-based method gives competitive results again compared with other methods in terms of SSIM indicator. The values obtained by all algorithms increase with the number of thresholds, which indicates that the segmented image is more similar to the original image in terms of brightness, contrast, and structural similarity. The experimental results in this section verify the remarkable performance of the proposed algorithm from another perspective.

### 6.6. Feature Similarity Index (FSIM)

Feature similarity index (FSIM) [54,55]: another measure of the image quality through evaluating the feature similarity between the original image and the segmented image:

(23) $$FSIM=\frac{\sum_{x\in \Omega }S_{L}\left(x\right)\times PC_{m}\left(x\right)}{\sum_{x\in \Omega }PC_{m}\left(x\right)}$$

where Ω represents the whole image pixel domain. $S_{L}\left(x\right)$ is a similarity score. $PC_{m}\left(x\right)$ denotes the phase consistency measure, which is defined as:

(24) $$PC_{m}\left(x\right)=\max\left(PC_{1}\left(x\right),PC_{2}\left(x\right)\right)$$

where $PC_{1}\left(x\right)$ and $PC_{2}\left(x\right)$ represent the phase consistency of two blocks, respectively:

(25) $$S_{L}\left(x\right)={\left[S_{PC}\left(x\right)\right]}^{\alpha }\cdot {\left[S_{G}\left(x\right)\right]}^{\beta }$$

(26) $$S_{PC}\left(x\right)=\frac{2PC_{1}\left(x\right)\times PC_{2}\left(x\right)+T_{1}}{PC_{1}^{2}\left(x\right)\times PC_{2}^{2}\left(x\right)+T_{1}}$$

(27) $$S_{G}\left(x\right)=\frac{2G_{1}\left(x\right)\times G_{2}\left(x\right)+T_{2}}{G_{1}^{2}\left(x\right)\times G_{2}^{2}\left(x\right)+T_{2}}$$

$S_{PC}\left(x\right)$ denotes the similarity measure of phase consistency. $S_{G}\left(x\right)$ denotes the gradient magnitude of two regions $G_{1}\left(x\right)$ and $G_{2}\left(x\right)$. α, β, $T_{1}$, and $T_{2}$ are all constants. The value of FSIM is also in the range [0,1], and a higher value shows better segmented image quality.

**Figure 9:**
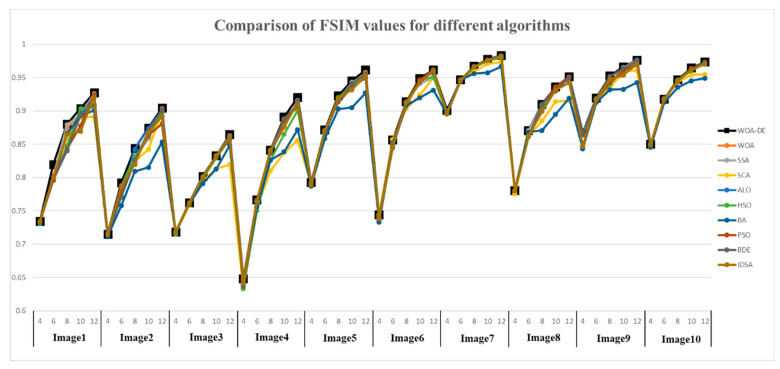
Comparison of FSIM values for different algorithms using Kapur’s entropy at 4, 6, 8, 10, and 12 levels.

On comparing the FSIM values, which are given in Table 3 and Figure 9, it can be observed that WOA-DE-based method again outperforms the other methods. The feature similarity between the original image and the segmented image is considered in this experiment to verify the quality of segmented image comprehensively. The relevant results indicate that the proposed method has a strong feature preserving ability as compared to other methods.

### 6.7. Convergence Performance

**Figure 10:**
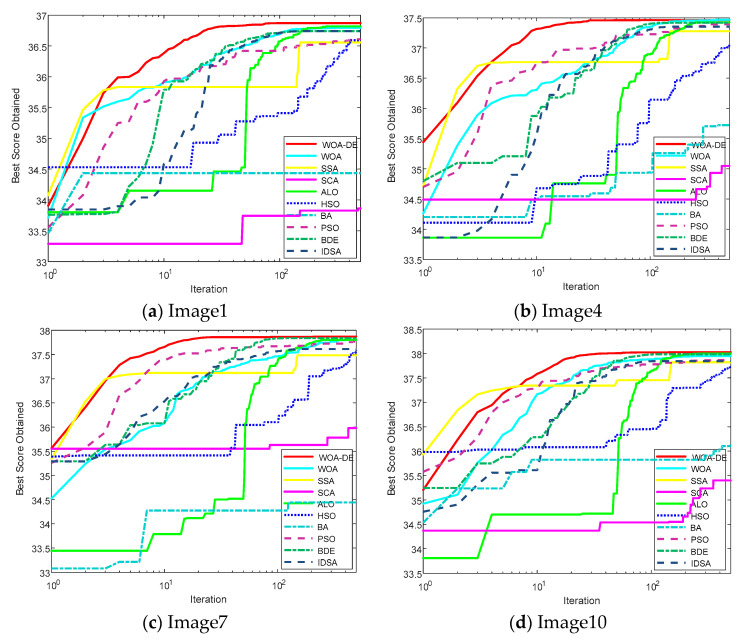
The convergence curves for fitness function using Kapur’s entropy method at 12 levels thresholding.

In this section, the convergence performance of all algorithms is evaluated and discussed in details. In order to reflect the performance of WOA-DE more intuitively, the convergence curves of Kapur’s entropy function (for *K* = 12) are shown in Figure 10. Four different images are selected for testing, namely “Image1”, “Image4”, “Image7”, and “Image10”. It can be found that the proposed algorithm outperforms other algorithms in general. In other words, the WOA-DE-based method gives higher position curves using Kapur’s entropy technique.

As discussed above, the main drawbacks of the standard WOA are premature convergence and unbalanced exploration-exploitation, which are clearly reflected in the curves. For example, under the circumstance of “Image1” segmentation, the objective function value of WOA is almost never updated after 100 iterations, while the optimal value obtained is not the best. This phenomenon illustrates the premature convergence shortcoming of WOA. However, the proposed WOA-DE algorithm gives the highest objective function value under the premise of ensuring the convergence speed. In fact, the remarkable performance of the proposed algorithm is not only reflected in the segmentation task of “Image1”, but also in other images. The experimental results in this section indicate that WOA-DE algorithm can better balance the exploration and exploitation, and the complex image segmentation tasks are also competent.

### 6.8. Computation Time

The average CPU time of different algorithms considering all cases is given in Table 4. It can be found from the table that HSO is the fastest among available methods, but the segmentation accuracy discussed above is not ideal. The standard WOA algorithm gives competitive results in some cases, and the proposed algorithm namely WOA-DE is slightly slower than the standard WOA. The reason for this phenomenon is the premature convergence of HSO algorithm, which cannot well balance exploration and exploitation. On the contrary, the WOA-DE algorithm combines the advantages of both WOA and DE, which determine the most appropriate threshold value, despite not being the fastest. To sum up, WOA-DE is a high-performance hybrid algorithm that improves segmentation precision while maintaining runtime.

### 6.9. Statistical Analysis

In this section, a non-parametric statistical test known as “Wilcoxon’s rank sum test” is used to evaluate the significant difference between algorithms [56]. The experiments are conducted 30 runs at significance level 5%. All experimental data obtained based on Kapur’s entropy are used for testing. The alternative hypothesis $\left(H_{1}\right)$ assumes that there is a significant difference between the two algorithms being compared. The null hypothesis $H_{0}$ considers that there is no significant difference between the algorithms. The results of the statistical experiments are given in Table 5.

It can be observed from the table that the *p*-values acquired are far less than 0.05. This promising result indicates that $H_{0}$ can be rejected in all cases and there is a significant difference between the proposed algorithm and other methods.

### 6.10. Comparison of Otsu and Kapur’s Entropy Methods

In order to obtain a simple and powerful technique for color image segmentation, an experiment of comparison between Otsu and Kapur’s entropy thresholding techniques based on WOA-DE is conducted in this section. More details of Otsu thresholding technique can be found in [11].

The PSNR, SSIM, and FSIM values obtained by WOA-DE-based method are given in Table 6. It can be seen that WOA-DE-based method using Kapur’s entropy gives higher values than using Otsu technique in general for PSNR values. However, the Otsu-based technique performs better when comparing SSIM values. Considering the FSIM indicator, these two thresholding techniques are equal. Precisely speaking, on comparing the PSNR values, the Otsu technique presents better results in 11 out of 50 cases (10 images and five thresholds), whereas, Kapur’s entropy technique gives better results in 39 out of 50 cases. Considering other two indicators, the Kapur’s entropy technique outperforms in 21 cases for SSIM and 25 cases for FSIM, while the Otsu technique outperforms in 29 cases for SSIM and 25 cases for FSIM. To sum up, the WOA-DE-based method through Otsu gives better results in 65 out of 150 cases (10 images, five thresholds, and three performance measures) and the WOA-DE-based method through Kapur’s entropy gives satisfactory results in 85 cases. To some extent, these satisfactory results prove that WOA-DE-based method using Kapur’s entropy is superior to the method using Otsu. However, as the no free lunch (NFL) theorem goes, there is no technique that can handle all image segmentation tasks [57]. Thus, the WOA-DE algorithm based on different thresholding techniques has potential in the field of color image segmentation, which may exhibit superior performance in some engineering problems that have not been solved so far.

### 6.11. Robustness Testing on Noisy Images

**Figure 11:**
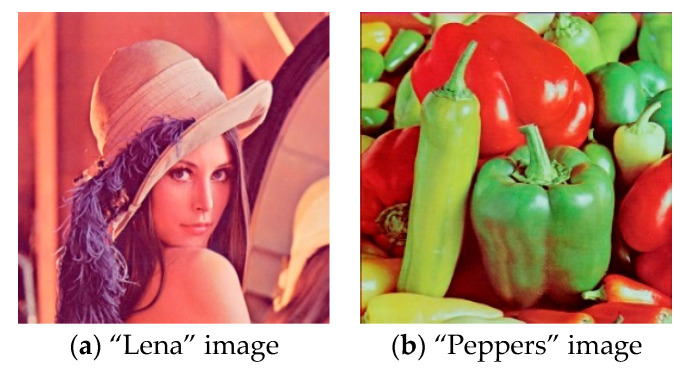
Original “Lena” and “Peppers” images from Berkeley Segmentation Dataset.

**Figure 12:**
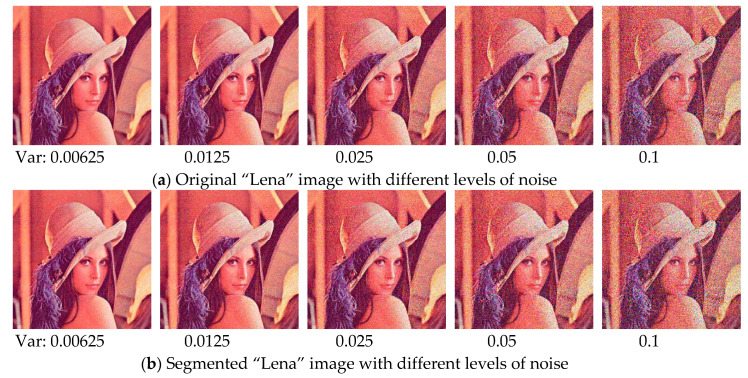
The original “Lena” image and the corresponding segmented results under various noise levels.

**Figure 13:**
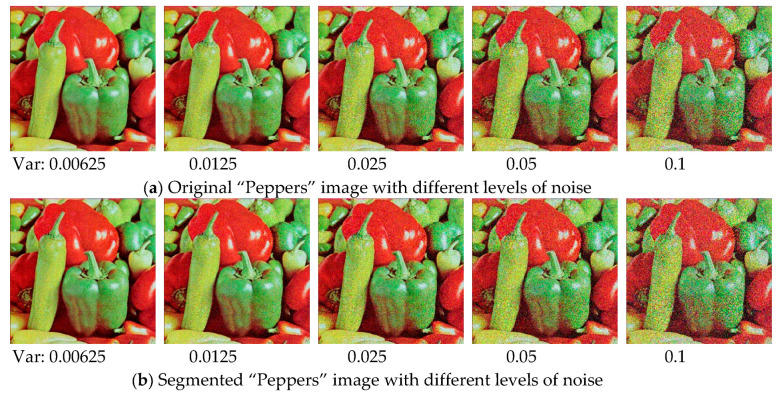
The original “Peppers” image and the corresponding segmented results under various noise levels.

**Figure 14:**
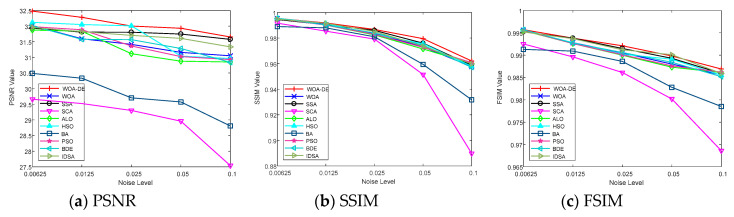
The value of various performance measures over “Lena” image with different levels of noise.

**Figure 15:**
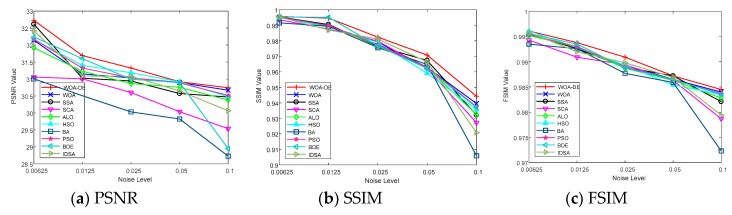
The value of various performance measures over “Peppers” image with different levels of noise.

In order to further investigate the performance of proposed algorithm, an experiment is conducted on two famous benchmark test images with various noise levels. “Lena” and “Peppers” images are used in this section (see Figure 11), which can be obtained from [58]. The mean value is fixed in this experiment, and the level of Gaussian noise is adjusted by setting the variance as 0.00625, 0.0125, 0.025, 0.05, and 0.1, respectively. The experiment is carried out at 12 threshold level, in which case the difference between algorithms is the most obvious. The relevant results are presented in Figure 12, Figure 13, Figure 14 and Figure 15. It can be observed from the results that the value of performance measures and quality of segmented image decrease with the increase of noise level, and the WOA-DE-Kapur outperforms other methods using Kapur entropy. The promising results indicate that the proposed technique has strong robustness, which can be competent for complex image segmentation tasks with noise.

### 6.12. Application in MR Image

In this section, the WOA-DE-Kapur-based multilevel thresholding technique is applied to the field of MR image segmentation. The purpose of this experiment is to investigate whether the proposed algorithm is capable of producing high quality segmented MR images. Two other threshold-based MR image segmentation techniques are used for comparison, namely the crow search algorithm-based method using minimum cross entropy thresholding (CSA-MCET) [59] and adaptive bacterial foraging algorithm-based method using Otsu (ABF-Otsu) [60]. The combination of thresholds (*K* = 2, 3, 4, and 5) selected is the same as that used by above two algorithms in their corresponding articles. Besides, the parameter values are set according to the original literature, except for the population size N set to 30 and the number of iterations $t_{\max}$ set to 500 for fair comparison. All experiments are performed 30 times to eliminate errors.

The experimental results are shown in three tables. Table 7 presents the optimal thresholds and PSNR values, Table 8 gives the SSIM and FSIM values, and Table 9 indicates the segmented images obtained by all methods. It can be found from these results that WOA-DE-Kapur method can determine more accurate thresholds compared to other methods. For quantitative analysis, the values of performance measures obtained by proposed method is higher, which indicate the better quality of segmented image. For visual analysis, WOA-DE-Kapur method gives more informative segmented MR images, and the details of image become more prominent as the number of thresholds increases.

Since the experiments of three methods are the same, it is necessary to carry out relevant statistical tests. In this section, Friedman test [61] and Wilcoxon’s rank sum test [56] are used as non-parametric statistical test to evaluate the performance of these methods considering 5% as significant level. Null hypothesis $\left(H_{0}\right)$ in Friedman test states equality of medians between the algorithms, and the alternative hypothesis $\left(H_{1}\right)$ indicates the difference. A more detailed description of Friedman test can be found in literature [62]. The results of the relevant statistical tests can be observed in Table 10 and Table 11. Table 10 presents the average rank and *p*-value of all algorithms at different threshold levels. As can be found, ABF-Otsu obtains the first rank for *K* = 3, and WOA-DE-Kapur provides the first rank in other cases. In other words, the proposed technique gives the best result in general. The *p*-value for all threshold levels is very small indicating the significant difference among available methods. Table 11 gives the result of Wilcoxon’s rank sum test. It can be observed that the *p*-value is less than 0.05 in most cases, which verifies the remarkable performance of WOA-DE-Kapur technique in a statistical and meaningful way.

## 7. Conclusions

In order to obtain an efficient technique for color image segmentation, an improved WOA-based method is introduced in this paper, which is known as WOA-DE. In the proposed algorithm, DE is adopted as a local search strategy with the purpose of enhancing exploitation capability. Compared to the traditional WOA, the WOA-DE algorithm can effectively avoid falling into a local optimum and prevent the loss of population diversity in the later iterations. A series of experiments have been conducted on various color images including natural images and satellite images. Seven meta-heuristic algorithms are utilized for comparison. The experimental results indicate that the proposed techniques outperform other methods in terms of average fitness values, standard deviation (STD), peak signal to noise ratio (PSNR), structural similarity index (SSIM), and feature similarity index (FSIM) as well as the Wilcoxon’s rank sum test. In addition, to give more convincing and reliable results, another thresholding technique namely Otsu is adopted for testing. The experimental results indicate that WOA-DE-based technique through Kapur’s entropy gives better results than using the Otsu technique in most cases. However, there is no technique that can handle all image segmentation tasks. Thus, it is necessary to introduce more and better techniques to meet the requirements of different image segmentation problems and this is also the motivation for our future research. The performance of some novel meta-heuristic algorithms will be evaluated in this domain, such as salp swarm algorithm, spotted hyena optimizer, emperor penguin optimizer, etc.

## Figures and Tables

**Table 1:** Parameters of the algorithms.

No.	Algorithm	Parameter Setting	Year	Reference
1	WOA-DE	CR=0.9(crossover rate),SF=0.5(scaling factor)	—	—
2	WOA OA	a∈[0,2]	2016	[21]
3	SSA	$c_{1}\in \left[0,2\right]$	2017	[42]
4	SCA	$r_{1}\in \left[0,2\right]$	2016	[43]
5	ALO	ω∈[2,6](constant)	2015	[44]
6	HSO	HMCR=0.9,PAR=0.3(pitch adjusting rate)	2001	[45]
7	BA	$r_{i}\in \left[0,1\right]\left(\mathrm{rate}\;\mathrm{of}\;\mathrm{pluse}\;\mathrm{emission}\right),A_{i}\in \left[1,2\right]\left(\mathrm{loudness}\;\mathrm{value}\right)$	2015	[46]
8	PSO	$c_{1}=c_{2}=2,w\in \left[0.4,0.9\right],v_{max}=25.5$	1995	[47]
9	BDE	a∈[0,1](beta distribution parameter)	2018	[49]
10	IDSA	—	2018	[50]

**Table 2:** The average fitness values and STD values obtained by all algorithms.

Measures	Image	*K*	WOA-DE	WOA	SSA	SCA	ALO	HSO	BA	PSO	BDE	IDSA
Mean	Image1	4	18.5843	18.5843	18.5843	18.5632	18.5843	18.5761	18.5818	18.5842	18.5843	18.5843
		6	23.8418	23.73	23.8408	23.479	23.8417	23.755	23.8085	23.8412	23.8115	23.8383
		8	28.5094	28.4605	28.5051	27.8225	28.4627	28.385	27.7631	28.4991	28.5118	28.5139
		10	32.8462	32.8432	32.8325	31.3685	32.8443	32.6682	32.0858	32.7519	32.8455	32.7269
		12	36.8534	36.7164	36.7269	34.5881	36.7313	36.6221	34.7641	36.696	36.7642	36.7764
	Image6	4	18.4839	18.4784	18.4817	18.4434	18.4836	18.4778	18.4745	18.4839	18.4816	18.4836
		6	24.0059	23.9988	23.9994	23.765	24.005	23.9687	23.9225	24.0015	24.0051	24.0059
		8	28.937	28.8743	28.8836	27.9973	28.9272	28.8508	28.2696	28.9293	28.9342	28.9196
		10	33.3483	33.3009	33.1851	31.8768	33.2743	33.0867	31.6321	33.3197	33.3079	33.2562
		12	37.3674	37.271	37.1046	35.8876	37.3246	36.8644	35.1068	37.1813	37.3553	37.2569
STD	Image1	4	**0**	**2.66 × 10^−5^**	**2.66 × 10^−5^**	**4.39 × 10^−3^**	**5.83 × 10^−5^**	**3.19 × 10^−3^**	**2.17 × 10^−3^**	**2.68 × 10^−5^**	2.47	**1.58 × 10^−1^**
		6	**3.25 × 10^−5^**	**1.61 × 10^−4^**	**9.58 × 10^−4^**	**7.45 × 10^−2^**	**2.95 × 10^−4^**	**1.97 × 10^−2^**	**5.39 × 10^−2^**	**4.33 × 10^−4^**	**8.41 × 10^−1^**	1.59
		8	**4.32 × 10^−4^**	**2.74 × 10^−2^**	**8.89 × 10^−3^**	**1.33 × 10^−1^**	**3.24 × 10^−2^**	**3.38 × 10^−2^**	**1.82 × 10^−1^**	**7.30 × 10^−3^**	1.3	**9.98 × 10^−1^**
		10	**3.38 × 10^−3^**	**5.36 × 10^−3^**	**3.90 × 10^−2^**	**2.67 × 10^−1^**	**4.91 × 10^−2^**	**3.24 × 10^−2^**	**1.51 × 10^−1^**	**3.34 × 10^−2^**	**7.76 × 10^−1^**	**7.27 × 10^−1^**
		12	**1.83 × 10^−2^**	**3.25 × 10^−2^**	**7.48 × 10^−2^**	**2.30 × 10^−1^**	**6.12 × 10^−2^**	**5.02 × 10^−2^**	**6.41 × 10^−1^**	**7.35 × 10^−2^**	**6.66 × 10^−1^**	**6.65 × 10^−1^**
	Image6	4	**3.91 × 10^−3^**	**7.91 × 10^−3^**	**4.81 × 10^−3^**	**1.24 × 10^−2^**	**8.29 × 10^−1^**	**5.49 × 10^−3^**	**4.60 × 10^−3^**	**3.93 × 10^−3^**	**6.96 × 10^−3^**	**2.92 × 10^−1^**
		6	**1.68 × 10^−2^**	**3.81 × 10^−3^**	**1.94 × 10^−2^**	**6.82 × 10^−2^**	**4.20 × 10^−3^**	**3.58 × 10^−2^**	**2.96 × 10^−2^**	**5.03 × 10^−3^**	4.66	1.75
		8	**1.57 × 10^−2^**	**2.64 × 10^−2^**	**1.64 × 10^−2^**	**1.60 × 10^−1^**	**2.48 × 10^−2^**	**4.37 × 10^−2^**	**3.94 × 10^−1^**	**6.14 × 10^−2^**	3.88	**9.82 × 10^−1^**
		10	**2.15 × 10^−2^**	**4.26 × 10^−2^**	**3.64 × 10^−2^**	**1.40 × 10^−1^**	**5.49 × 10^−2^**	**2.50 × 10^−2^**	**4.03 × 10^−1^**	**3.55 × 10^−2^**	2.28	**8.98 × 10^−1^**
		12	**1.53 × 10^−2^**	**3.02 × 10^−2^**	**9.90 × 10^−2^**	**2.85 × 10^−1^**	**2.52 × 10^−2^**	**6.41 × 10^−2^**	**3.37 × 10^−1^**	**3.43 × 10^−2^**	1.67	1.25

**Table 3:** The PSNR, SSIM, and FSIM values obtained by all algorithms under different threshold levels.

Measures	Image	*K*	WOA-DE	WOA	SSA	SCA	ALO	HSO	BA	PSO	BDE	IDSA
PSNR	Image2	4	18.6558	18.6558	18.6558	18.6533	18.6558	18.5722	18.4352	18.6558	18.6452	18.6558
		6	22.2481	20.8588	21.3402	21.5799	21.3148	20.861	20.2596	21.7136	20.8588	20.9995
		8	24.8821	23.1744	23.6373	23.4877	24.1624	24.5724	23.1158	23.372	23.5863	23.5837
		10	27.9116	25.3956	25.9502	27.0446	27.7051	25.3211	25.1289	25.3938	25.87	25.9861
		12	29.8805	29.8395	29.6719	28.6767	29.4309	26.3023	29.2218	29.34	29.0663	29.2001
	Image7	4	23.2367	22.947	22.9286	22.9305	23.0442	22.9765	22.923	22.982	22.982	22.9122
		6	26.6481	26.5553	26.5205	26.5953	26.656	26.4685	26.5963	26.6156	26.527	26.5732
		8	29.1886	29.1004	28.9405	27.8606	29.132	29.063	27.9378	29.0088	29.1151	29.0763
		10	31.2154	30.9433	31.1186	29.6665	30.9579	30.64	28.3997	30.9199	31.0374	31.169
		12	32.7203	32.6538	31.7022	30.101	32.6566	31.6295	28.988	32.7035	32.6774	32.6603
SSIM	Image2	4	0.5266	0.5266	0.5266	0.5253	0.5186	0.5212	0.5212	0.5266	0.5242	0.5266
		6	0.652	0.6103	0.617	0.6105	0.6192	0.6379	0.5864	0.6332	0.6103	0.6197
		8	0.7361	0.6944	0.7052	0.6976	0.7281	0.7224	0.7094	0.6978	0.7004	0.6963
		10	0.8064	0.7551	0.7608	0.7701	0.7996	0.7534	0.7247	0.7594	0.7705	0.7733
		12	0.8505	0.8484	0.8432	0.7859	0.8411	0.8463	0.8182	0.8367	0.8483	0.8269
	Image7	4	0.8494	0.8414	0.8407	0.8419	0.8456	0.8405	0.8382	0.8416	0.8416	0.8399
		6	0.9136	0.9097	0.907	0.9086	0.9115	0.9091	0.9098	0.9087	0.9091	0.9079
		8	0.9422	0.9409	0.9392	0.9254	0.9415	0.9412	0.9241	0.9404	0.9416	0.9418
		10	0.9648	0.9608	0.9603	0.946	0.9587	0.9552	0.9255	0.9624	0.9633	0.9637
		12	0.9726	0.9715	0.9624	0.9543	0.9724	0.963	0.9338	0.9718	0.9724	0.9725
FSIM	Image2	4	0.7151	0.7151	0.7151	0.7149	0.7151	0.7117	0.7115	0.7151	0.7142	0.7151
		6	0.7921	0.7707	0.7723	0.7708	0.7711	0.7876	0.7577	0.7799	0.7707	0.7866
		8	0.8435	0.8257	0.8289	0.8246	0.8426	0.8313	0.8093	0.824	0.8256	0.8198
		10	0.8745	0.8617	0.8582	0.8423	0.8738	0.8616	0.8153	0.864	0.8686	0.8682
		12	0.9041	0.9036	0.9005	0.8987	0.9007	0.8978	0.8531	0.8806	0.9022	0.8915
	Image7	4	0.9012	0.8964	0.8959	0.8965	0.8991	0.8968	0.8953	0.8972	0.8972	0.8956
		6	0.9469	0.9445	0.946	0.9458	0.9467	0.9449	0.9468	0.9465	0.9453	0.9464
		8	0.9671	0.9666	0.9658	0.9591	0.9665	0.9659	0.9559	0.9658	0.9666	0.966
		10	0.9773	0.9758	0.9752	0.971	0.9759	0.9754	0.9574	0.9765	0.9761	0.9763
		12	0.9834	0.9824	0.9799	0.9729	0.9829	0.9802	0.9664	0.9828	0.9825	0.9826

**Table 4:** The average computation time (s) considering all images under different threshold levels.

*K*	WOA-DE	WOA	SSA	SCA	ALO	HSO	BA	PSO	BDE	IDSA
4	1.40087	1.047	1.49062	1.49438	7.8046	1.03739	1.97122	1.70887	2.21335	1.41216
6	1.55259	1.14527	1.63902	1.62171	9.66773	1.10338	2.08452	1.88491	2.40389	1.5397
8	1.67041	1.18449	1.72857	1.6764	12.09074	1.18478	2.31804	1.99103	2.48257	1.56885
10	1.74287	1.24446	1.79294	1.86849	15.31865	1.23933	2.36836	2.13532	2.58595	1.67435
12	1.88104	1.39335	1.95442	1.98369	17.19651	1.30339	2.513	2.23487	2.74791	1.70745

**Table 5:** Wilcoxon’s rank sum test results.

Comparison	*p*-Value
WOA-DE versus WOA	2.3197 × 10^−4^
WOA-DE versus SSA	9.0193 × 10^−8^
WOA-DE versus SCA	6.8546 × 10^−7^
WOA-DE versus ALO	4.2264 × 10^−10^
WOA-DE versus HSO	7.6791 × 10^−7^
WOA-DE versus BA	3.2115 × 10^−9^
WOA-DE versus PSO	7.6473 × 10^−8^
WOA-DE versus BDE	4.5474 × 10^−5^
WOA-DE versus IDSA	7.0546 × 10^−4^

**Table 6:** Comparison of Kapur’s entropy and Otsu methods based on WOA-DE algorithm.

Images	*K*	PSNR		SSIM		FSIM	
		Otsu	Kapur	Otsu	Kapur	Otsu	Kapur
Image1	4	20.3428	17.7781	0.5798	0.4681	0.7771	0.734
	6	22.6702	24.977	0.6815	0.6559	0.8458	0.8197
	8	24.0516	28.6092	0.7446	0.8033	0.9122	0.8798
	10	25.2164	30.6687	0.7898	0.833	0.9225	0.9039
	12	26.1897	32.2054	0.8059	0.8672	0.926	0.9271
Image2	4	18.459	18.6558	0.608	0.5266	0.7582	0.7151
	6	20.9182	22.2481	0.7095	0.652	0.8245	0.7921
	8	24.5622	24.8821	0.8164	0.7361	0.8684	0.8435
	10	25.7585	27.9116	0.8421	0.8064	0.8878	0.8745
	12	28.4144	29.8805	0.8964	0.8505	0.917	0.9041
Image3	4	17.5776	20.8247	0.6971	0.7109	0.6972	0.7182
	6	22.7555	23.6059	0.7431	0.7592	0.7469	0.7619
	8	27.8967	26.0132	0.7948	0.8036	0.795	0.8017
	10	29.5405	29.0184	0.8341	0.8433	0.8322	0.8329
	12	31.6891	32.6886	0.8633	0.8729	0.8619	0.8646
Image4	4	19.0015	23.013	0.6151	0.612	0.7012	0.6484
	6	24.4296	26.9872	0.7631	0.7188	0.8129	0.7665
	8	27.9781	29.7682	0.8434	0.7953	0.8793	0.8415
	10	32.0713	31.7603	0.8888	0.8456	0.9193	0.8907
	12	33.9227	33.096	0.9194	0.8767	0.9431	0.9203
Image5	4	23.4509	23.4495	0.8082	0.7231	0.8469	0.7925
	6	27.2396	27.2417	0.8948	0.8286	0.9142	0.8716
	8	29.6073	29.5852	0.926	0.8903	0.9415	0.9229
	10	31.5024	31.4704	0.9345	0.919	0.9585	0.9452
	12	32.9105	32.782	0.9457	0.9429	0.9672	0.9614
Image6	4	19.2192	20.597	0.6626	0.5995	0.7712	0.7443
	6	23.4934	25.4883	0.8061	0.7587	0.8673	0.8562
	8	27.6467	28.6898	0.8732	0.8427	0.9192	0.9136
	10	29.7289	31.292	0.9104	0.9002	0.9416	0.9489
	12	32.0406	32.7058	0.9384	0.9227	0.9599	0.9615
Image7	4	18.9474	23.2367	0.7898	0.8494	0.848	0.9012
	6	23.6742	26.6481	0.8938	0.9136	0.9198	0.9469
	8	26.8294	29.1886	0.9383	0.9422	0.9513	0.9671
	10	30.559	31.2154	0.9626	0.9648	0.9728	0.9773
	12	32.9021	32.7203	0.9781	0.9726	0.9828	0.9834
Image8	4	20.3695	19.4801	0.5372	0.4881	0.786	0.7807
	6	23.4982	25.5717	0.6365	0.7043	0.8643	0.8705
	8	25.5399	27.8173	0.7326	0.7823	0.9007	0.9102
	10	27.2326	30.6727	0.8174	0.8479	0.9228	0.9361
	12	30.4945	32.0442	0.8483	0.8849	0.943	0.9514
Image9	4	20.5858	22.1696	0.6759	0.6581	0.8498	0.8671
	6	25.1403	26.3449	0.7465	0.7492	0.9174	0.9197
	8	28.672	29.2954	0.7938	0.8082	0.9476	0.9524
	10	30.9026	31.126	0.8711	0.8716	0.9664	0.9661
	12	32.5855	32.9878	0.9012	0.8757	0.9761	0.9764
Image10	4	20.2121	22.6128	0.7399	0.7551	0.8312	0.8499
	6	24.9168	27.0397	0.8128	0.8355	0.9075	0.9179
	8	29.1254	29.5441	0.8865	0.8649	0.9503	0.947
	10	30.9532	31.447	0.9196	0.8774	0.9641	0.9645
	12	32.5129	32.8351	0.9284	0.8923	0.9729	0.9734
Rank		2(11)	1(39)	2(29)	1(21)	1(25)	1(25)

**Table 7:** Comparison of Optimal threshold and PSNR value obtained by WOA-DE-Kapur, ABF-Otsu, and CSA-MCET.

Images	*K*	Optimal Threshold Value			PSNR		
		WOA-DE-Kapur	ABF-Otsu	CSA-MCET	WOA-DE-Kapur	ABF-Otsu	CSA-MCET
Slice20	2	94 167	28 97	13 84	16.8586	16.524	15.9746
	3	9 118 219	29 87 151	18 64 134	23.9008	23.1061	22.4605
	4	8 29 129 210	7 53 100 153	16 64 98 147	24.6228	25.4972	24.3967
	5	16 36 94 171 211	21 54 98 156 190	3 40 61 113 150	30.4912	27.3411	28.5034
Slice24	2	111 182	48 145	19 118	19.7345	21.0839	20.8004
	3	34 117 182	40 108 172	7 56 136	23.4428	22.9913	23.5030
	4	17 73 129 193	23 70 118 182	6 50 101 161	26.7848	26.2061	24.7095
	5	14 70 115 165 210	20 63 102 143 196	4 27 66 111 170	28.9204	28.3318	25.3871
Slice28	2	114 179	52 151	20 121	19.6991	18.6884	19.1865
	3	20 81 156	46 110 175	7 56 139	24.8983	24.3616	23.7032
	4	22 78 137 192	27 76 126 187	6 48 103 161	26.9455	27.0419	25.8075
	5	13 72 117 157 203	23 68 109 149 203	6 36 74 115 174	29.6822	29.1884	28.1382
Slice32	2	115 175	53 159	20 137	23.3496	22.888	22.6576
	3	16 76 143	50 120 189	8 54 148	24.711	23.2735	25.9537
	4	16 74 131 186	21 70 122 191	7 52 107 172	27.5852	27.958	27.947
	5	18 71 118 162 205	19 63 105 147 206	3 28 67 116 180	29.7914	28.6183	29.598
Rank		—	—	—	1(10)	2(4)	3(2)

**Table 8:** Comparison of SSIM and FSIM value obtained by WOA-DE-Kapur, ABF-Otsu, and CSA-MCET.

Images	*K*	SSIM			FSIM		
		WOA-DE-Kapur	ABF-Otsu	CSA-MCET	WOA-DE-Kapur	ABF-Otsu	CSA-MCET
Slice20	2	0.7923	0.7726	0.7882	0.8743	0.8565	0.8421
	3	0.8784	0.8061	0.8811	0.9411	0.9305	0.9594
	4	0.9225	0.8408	0.9208	0.9608	0.9614	0.9599
	5	0.9435	0.8862	0.9249	0.9882	0.9674	0.9723
Slice24	2	0.6809	0.7886	0.7865	0.7772	0.8178	0.8117
	3	0.8391	0.8318	0.8343	0.8686	0.8660	0.8394
	4	0.8791	0.8770	0.8742	0.9081	0.9026	0.8944
	5	0.9015	0.8959	0.8997	0.9277	0.9253	0.9099
Slice28	2	0.7832	0.7678	0.7792	0.813	0.8394	0.8274
	3	0.8365	0.8238	0.8275	0.8849	0.8846	0.8585
	4	0.8672	0.8687	0.8691	0.9084	0.9136	0.9156
	5	0.8993	0.8937	0.9010	0.9371	0.9355	0.9366
Slice32	2	0.8123	0.7973	0.7862	0.8617	0.8388	0.8589
	3	0.8465	0.832	0.8513	0.8864	0.8943	0.9009
	4	0.8794	0.8824	0.8784	0.9199	0.9271	0.9275
	5	0.9023	0.8705	0.8991	0.9477	0.9237	0.9347
Rank		1(10)	3(2)	2(4)	1(9)	3(3)	2(4)

**Table 9:** The segmented MRI for different algorithms at 2, 3, 4, and 5 levels.

*K*	WOA-DE-Kapur	ABF-Otsu	CSA-MCET	WOA-DE-Kapur	ABF-Otsu	CSA-MCET
	Slice20			Slice24		
2	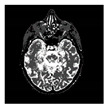	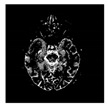	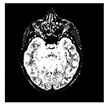	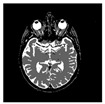	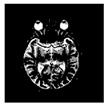	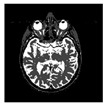
3	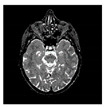	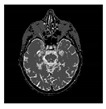	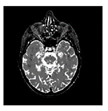	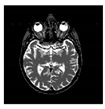	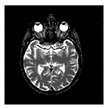	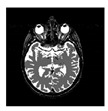
4	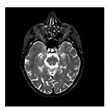	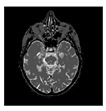	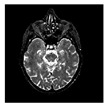	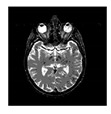	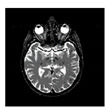	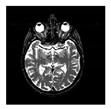
5	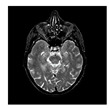	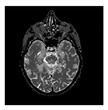	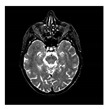	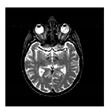	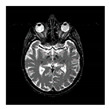	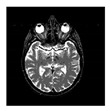
***K***	**Slice28**			**Slice32**		
2	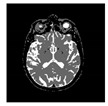	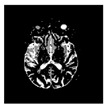	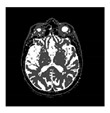	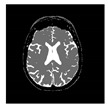	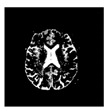	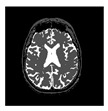
3	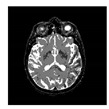	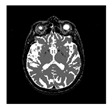	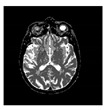	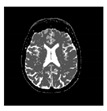	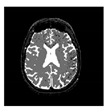	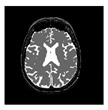
4	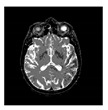	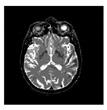	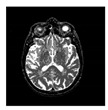	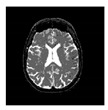	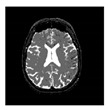	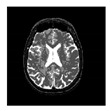
5	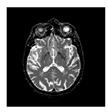	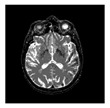	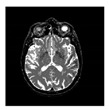	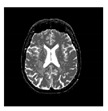	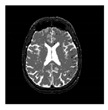	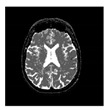

**Table 10:** Friedman test for WOA-DE-Kapur, ABF-Otsu, and CSA-MCET on MR images.

*K*	Average Rank			*p*-Value
	WOA-DE-Kapur	ABF-Otsu	CSA-MCET	
2	1.6667	2.0000	2.3333	2.2619 × 10^−7^
3	1.5833	2.5833	1.8333	1.1603 × 10^−8^
4	2.0000	1.6667	2.3333	7.2217 × 10^−9^
5	1.0833	2.7500	2.1667	5.3467 × 10^−9^

**Table 11:** Wilcoxon’s rank sum test for WOA-DE-Kapur, ABF-Otsu, and CSA-MCET on MR images.

*K*	WOA-DE-Kapur vs. ABF-Otsu		WOA-DE-Kapur vs. CSA-MCET	
	*p*-Value	*h*	*p*-Value	*h*
2	< 0.05	1	< 0.05	1
3	< 0.05	1	0.0926	0
4	< 0.05	1	< 0.05	1
5	< 0.05	1	< 0.05	1
